# Effect of Maternally Derived Anti-protein and Anticapsular IgG Antibodies on the Rate of Acquisition of Nasopharyngeal Carriage of *Pneumococcus* in Newborns

**DOI:** 10.1093/cid/cix742

**Published:** 2017-08-17

**Authors:** John Ojal, David Goldblatt, Caroline Tigoi, J Anthony G Scott

**Affiliations:** 1 KEMRI-Wellcome Trust Research Programme, Centre for Geographic Medicine-Coast, Kilifi, Kenya; 2 Department of Infectious Disease Epidemiology, Faculty of Epidemiology and Population Health, London School of Hygiene and Tropical Medicine, London, United Kingdom; 3 Great Ormond Street Institute of Child Health, University College, London, United Kingdom

**Keywords:** antibodies, pneumococcus, nasopharyngeal carriage, Kenya

## Abstract

**Background:**

In developing countries, introduction of pneumococcal conjugate vaccine has not eliminated circulation of vaccine serotypes. Vaccinating pregnant mothers to increase antibody concentrations in their newborn infants may reduce the acquisition of pneumococcal carriage and subsequent risk of disease. We explored the efficacy of passive immunity, attributable to anti-protein and anticapsular pneumococcal antibodies, against acquisition of carriage.

**Methods:**

We examined the rate of nasopharyngeal acquisition of pneumococci in the first 90 days of life associated with varying anticapsular and anti-protein antibody concentrations in infant cord/maternal venous blood in Kilifi, Kenya. We used multivariable Cox proportional hazard models to estimate continuous functions relating acquisition of nasopharyngeal carriage to the concentration of maternally derived antibody.

**Results:**

Cord blood or maternal venous samples were collected from 976 mother-infant pairs. Pneumococci were acquired 561 times during 33,905 person-days of follow-up. Increasing concentrations of anti-protein antibodies were associated with either a reduction (PhtD1, PspAFam2, Spr0096, StkP) or, paradoxically, an increase (CbpA, LytC, PcpA, PiaA, PspAFam1, RrgBT4) in acquisition rate. We observed a nonsignificant reduction in the incidence of homologous carriage acquisition with high concentrations of maternally derived anticapsular antibodies to 5 serotypes (6A, 6B, 14, 19F, and 23F).

**Conclusion:**

The protective efficacy of several anti-protein antibodies supports the strategy of maternal vaccination to protect young infants from carriage and invasive disease. We were not able to demonstrate that passive anticapsular antibodies were protective against carriage acquisition at naturally occurring concentrations though it remains possible they may do so at the higher concentrations elicited by vaccination.

## Teaser

A strategy of maternal vaccination to protect newborns against carriage is likely to be successful if vaccine formulation is focused on enhancing specific anti-protein antibodies, or if the strategy induces very high anticapsular antibody concentrations, above natural maternally derived concentrations.

Among infants in low-income countries, pneumococcal carriage is acquired rapidly. In Kenya, in the prevaccination era, more than 80% of newborns acquired nasopharyngeal carriage by the age of 90 days [1]. The median time to colonization was 45 days in Thailand [2], whereas in India 54% of infants aged 2 months carried pneumococci [3]. Colonization is an essential step in the pathway to invasive pneumococcal disease (IPD) [4]. In a prevaccine surveillance exercise in Kilifi, Kenya, 15% of IPD episodes occurred in the first 2 months of life [5].

Pneumococcal conjugate vaccine (PCV) has been introduced in many low-income countries in schedules where infants are first vaccinated at either 6 weeks or 2 months of age [6]. In addition to direct protection of the vaccinated infant, the vaccine provides herd protection to unvaccinated individuals by interrupting transmission [7]. However, in contrast to developed countries [8], herd protection has not eliminated the circulation of vaccine serotypes in low-income countries, and evidence from Kenya [9] and The Gambia [10] suggests that the prevalence of vaccine serotypes remains relatively high several years after vaccine introduction.

This justifies the evaluation of maternal or newborn vaccination as strategies to protect young infants. Newborn vaccination with the 7-valent PCV was safe and immunogenic in Kenya and Papua New Guinea [11, 12]. In a review of studies of maternal vaccination, no safety concerns were reported with the 23-valent pneumococcal polysaccharide vaccine (PPV-23) or in the only study of maternal vaccination with a 9-valent PCV [13, 14]. Vaccination increased the passive transfer of antibodies to newborns, more so with the PCV [13]. Protein and whole cell pneumococcal vaccines, currently in clinical development, are designed to protect recipients against all serotypes of pneumococcus [15] and could be used, potentially, to protect young infants through newborn or maternal vaccination.

The correlates of protection (CoP) against invasive pneumococcal disease (IPD) have been established [16]. Attempts to derive CoP for vaccine-induced protection against carriage have not provided a clear-cut threshold [17, 18]. In addition, previous studies of this relationship have been constrained by several methodological limitations.

Most studies, for example, relating newborn colonization to maternally derived antibodies, with [19, 20] or without maternal vaccination [21–23], have failed to account for the colonization status of the mother at birth. Children born to carrier mothers have a higher risk of infection by the mother and are also likely to receive higher antibody concentrations by passive transfer, thus confounding the relationship between antibody concentration and carriage.

Second, the ascertainment of carriage acquisition in infants has been relatively insensitive. The earliest swabs were obtained at no younger than 1 month of age [20–25], and the period between subsequent swabs was also at least a month in all of the studies. Third, previous studies have been undermined by the relatively small sample sizes, which limits the power to detect modest protective efficacies. Clinical trials of maternal vaccination studied samples of both vaccinated and control children ranging in size from 46 to 437 infants [14], and studies without maternal vaccination had sample sizes in the range 51–310 [21–23, 25]. Finally, only one previous study has measured both anticapsular and anti-protein antibodies in the mother-infant pairs, and even here they were assessed independently [25].

Understanding the association between maternally derived antibodies and the rate of carriage acquisition for a panel of pneumococcal proteins and capsular polysaccharides is likely to guide antigen selection in future maternal/newborn immunization strategies. We aimed to characterize this association using a study where the carriage status of the mother at birth is already known; the ascertainment of carriage in the infant begins early and recurs frequently; the study population is of sufficient size to detect moderate associations; and the effects of anti-protein and anticapsular antibodies are analyzed simultaneously to determine the independent protective efficacies of each.

## METHODS

### Data

The study population and design have been described in detail elsewhere [1]. The study was conducted before the introduction of PCV vaccination in Kenya. Briefly, we collected nasopharyngeal swab specimens from participating newborns, aged at most 7 days, twice weekly for 2 weeks, and weekly thereafter until a pneumococcus was cultured from an infant’s swab or until 13 weeks after study entry, whichever was sooner. Mothers were swabbed at the time of birth and monthly thereafter. Cord blood was collected at the time of birth if the delivery took place at the hospital. Venous blood was collected from the mother if the child was born at home and reported to hospital within 7 days.

The environmental risk factors for carriage ascertained were: sex, mother’s HIV status, history of cough, history of coryza, observed cough, observed coryza, observed runny nose and breastfeeding status. At the household level we ascertained: type of fuel used for cooking, number of siblings aged <10 years, number of other children aged <10 years, number of adults, number of smokers, and number of carers.

### Laboratory Methods

A direct binding electrochemiluminescence-based multiplex assay [26] was used to measure serum immunoglobulin G (IgG) antibodies to 27 pneumococcal protein antigens. Pneumococcal reference serum 007sp was used as a standard and assigned a value of 1000 arbitrary units for each antigen [27]. Antibody levels in serum samples were expressed as concentrations with reference to the amount in 007sp.

We used enzyme-linked immunosorbent assay (ELISA) to analyze serum samples for antibodies to 6A, 6B, 14, 19F, and 23F capsular polysaccharides as described previously [28]. The assays were done at the World Health Organization (WHO) reference laboratory for pneumococcal serology, University College London Institute of Child Health, UK. These 5 serotypes were chosen because they were the serotypes most frequently acquired in the study.

### Statistical Analysis

Univariable Cox proportional hazard (Cox PH) models were fitted to assess the relationship between the hazard of acquisition of carriage and anti-protein antibody concentrations for each of the 27 proteins. Nonlinear effects of antibodies were modeled using restricted cubic splines [29]. The univariable Cox PH models were also fitted for homologous carriage against serotype-specific anticapsular antibody concentrations. Subjects were censored upon acquiring any pneumococcal serotype. For serotype-specific analyses this introduces a competing-risk scenario. Therefore, instead of estimating the standard hazard rates, we estimated the cause-specific hazard rates [30].

The principal problem with serotype-specific analyses is power, because only a subset of all pneumococcal acquisitions is used. We replicated timespan records for each individual 4 times resulting in 5 copies. We then associated each replicate record with a standardized log IgG concentration, as well as an indicator for acquisition, for each of the 5 serotypes. The log IgG concentrations were standardized using serotype-specific means and standard deviations. This restructured data set allowed us to estimate the impact of anticapsular antibodies on the acquisition of any of the 5 serotypes in a single model. We calculated cluster robust standard errors to account for the correlation introduced by replication.

We used the least absolute shrinkage and selection operator (LASSO) penalty to select anti-protein antibodies and environmental risk factors to retain in multivariable Cox PH models. We assumed that these factors were independent of serotype and therefore examined their effect by fitting a penalized Cox PH model with acquisition of any pneumococcus as outcome. The LASSO procedure shrinks the coefficients of the less relevant variables to zero and results in a set of variables that have optimal predictive value. Simulations indicate that the LASSO procedure can be more accurate than stepwise selection [31]. In the presence of strong correlations between candidate predictors, the LASSO may not be consistent in variable selection [32]. Consistency here refers to a higher ability to recover the correct model with growing number of observations. To achieve consistency we used an ensemble voting approach [33]. This involved fitting the penalized model to the observed data set and across 200 bootstrap-resampled data sets, and using this pool of estimated coefficients to vote on which variables to include in the model. A variable was selected if its coefficient was not reduced to zero in more than 50% of the resampled data sets, a threshold that is a balance between being too restrictive or too lenient.

We estimated nonlinear effects on acquisition of any serotype of each selected anti-protein antibody adjusting for selected environmental variables and remaining selected protein variables. All the Cox PH models of the effect of anticapsular IgG on the hazard of serotype-specific acquisition were adjusted for the selected environmental and protein variables. In these models, we entered the anticapsular or anti-protein IgG coefficient of interest unpenalized unlike the rest of the predictors used in adjustment. We also estimated nonlinear effects on acquisition of the 5 serotypes of each selected protein antibody adjusting for selected environmental variables, the remaining selected protein variables and the standardized anticapsular antibodies. Anticapsular concentrations below the minimum detectable limit of 0.15 mcg/mL were imputed as half the value, that is, 0.15/2 = 0.075. All anti-protein and anticapsular concentrations were log-transformed before analysis.

## RESULTS

### Acquisition Rates

Cord or venous blood samples were collected from 976 newborns; 342 (35%) of these were venous blood. The newborns were followed for a total of 33,905 days resulting in 561 acquisitions of pneumococcus; 218 (39%) of these were for serotypes 6A, 6B, 14, 19F, and 23F. The rate of acquisition per infant per 1000 days ranged from 0.77 for serotype 14 to 2.18 for serotype 19F (Table 1).

**Table 1.** Acquisitions Rates of Serotypes

Serotype	Acquisitions	Incidence (per 1000 days)	95% CI	Incidence in larger cohort^a^ (per 1000 days)
6A	45	1.33	0.97–1.77	1.49
6B	38	1.12	0.79–1.54	1.26
14	26	0.77	0.50–1.12	0.79
19F	74	2.18	1.71–2.74	2.54
23F	35	1.03	0.72–1.44	1.24

The total time at risk was 33,905 person-days.

Abbreviation: CI, confidence interval.

^**a**^The incidence estimated from the cohort of 1400 that included all newborns; those who gave and those who did not give serum samples.

### Univariable Analyses

Higher levels of anti-protein IgG were associated with reduction in acquisition of any serotype for a number of proteins; nearly monotonic relations between acquisition hazard and IgG level were estimated for PhtD1, PhtD2, PhtE, and StkP (*P* > .15 for each). Higher anti-protein antibodies to PiaA, RrgBT4, RrgB6B, and RrgB23F were associated with increased acquisition of carriage (*P* < .033 for each) (Figure S1).

**Figure 1.**
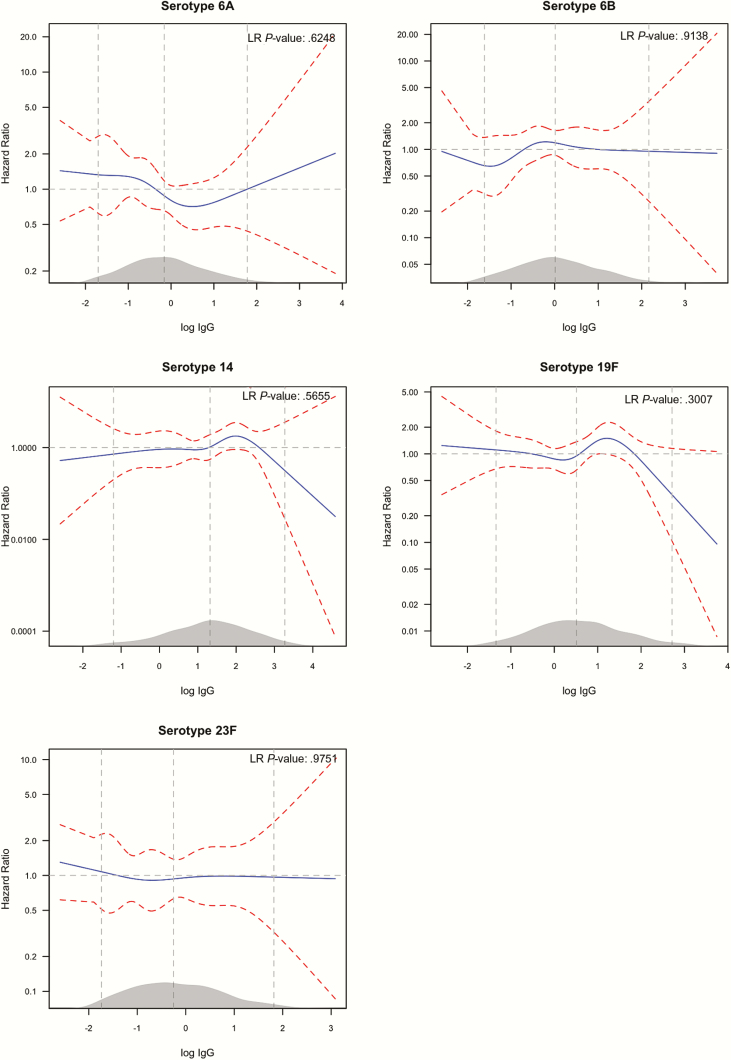
Univariable analysis of the effect of anti-capsular IgG on serotype-specific carriage acquisition rate. The figure shows the relative hazard of acquisition (blue solid line) as a function of log IgG concentration (x-axis) for each serotype labeled above the graph. The hazard at each level of log IgG is relative to the mean log IgG. The red dashed lines are the 95%CI bounds of the hazard ratio. The 3 vertical (gray) lines mark the 2.5th, 50th and 97.5th percentiles of the distribution of log IgG whose density is shown in grey on the x-axis. The likelihood ratio (LR) test *P*-value compares a model with and that without the log IgG concentration variable, thus indicating the overall significance of antibody concentration. The point-wise confidence intervals in the figures, at the point where the upper bound descends below the hazard ratio of 1, can be used to mark log IgG levels which results in significant reduction in carriage rates compared to typical (mean) log IgG values. Abbreviation: IgG, immunoglobulin G.

**Figure 2.**
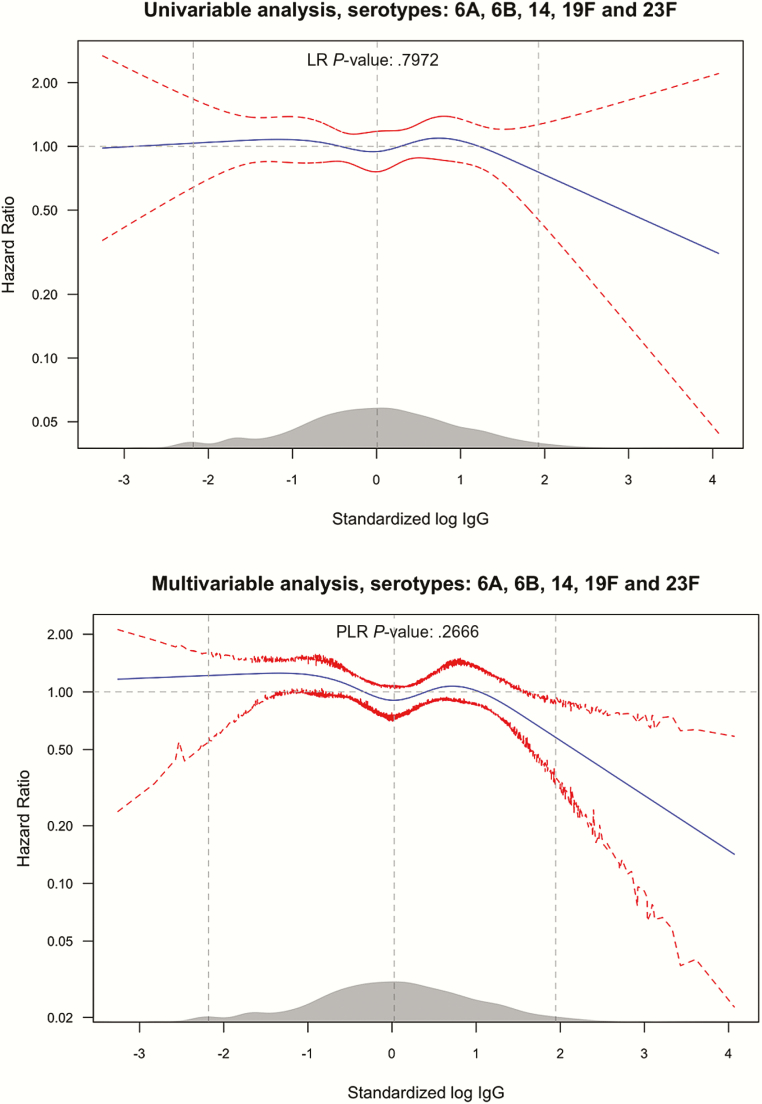
Univariable (upper panel) and Multivariable (lower panel) analysis of the effect of anti-capsular IgG concentration on carriage acquisition rates of any of the serotypes: 6A, 6B, 14, 19F and 23F. The figure follows the convention described in the legend for Figure 1. The 95% CIs for the multivariable analysis are percentile-based and computed from 200 cluster bootstrap (clustered on subject/individual) resamples of the data. PLR stands for penalized likelihood ratio test, comparing a model with the log IgG concentration variable and one without. Abbreviations: CI, confidence interval; IgG, immunoglobulin G.

The serotype-specific hazard of acquisition as a function of homologous log IgG concentration did not show a monotonically decreasing trend with higher concentrations (Figure 1). For serotypes 14 and 19F, there was favorable reduction in acquisition hazard at higher IgG levels. In the joint analysis combining all 5 serotypes, higher anticapsular IgG concentrations, on the extreme right of the distribution of IgG concentrations, were associated with a reduction in acquisition (Figure 2); however, we did not find evidence of an association across the whole range of values (*P* = .797). The point-wise confidence intervals (CIs) in the figures, at the upper bound descends below the hazard ratio (HR) of 1; this can be used to mark log IgG levels, which result in a significant reduction in carriage rates compared to typical (mean) log IgG values. In the univariable analysis, the upper limits of the CIs are all above a HR of 1.

### Multivariable Analyses

Table 2 shows the set of environmental and anti-protein antibody variables selected by the lasso procedure. In the set of candidate environmental predictors, sex, history of cough, observed cough, observed runny nose, and breastfeeding status were voted out of the model. Among the 27 candidate anti-protein antibody predictors, 17 were voted out. Except for antibodies to PhtD1, PspAFam2, StkP, and Spr0096, in which higher concentrations were associated with reduction in carriage acquisition, higher IgG levels for the remaining proteins were associated with increased acquisition (Table 2). The group of newborns who were born in hospital had a lower rate of pneumococcal acquisition (18% lower) compared to newborns who were born at home (Table 2).

**Table 2.** Adjusted Hazard Ratios Quantifying the Association of Acquisition of Any Pneumococcal Serotypes With Selected Environmental and Anti-protein Antibody Variables

Variable	Hazard ratio^a^	95% CI
Coryza observed at last visit	1.36	1.03–1.60
History of coryza at last visit	1.20	0.87–1.63
Runny nose at last visit	1.17	0.96–1.51
Fuel used for cooking		
Firewood	Ref.	
Charcoal	0.61	0.37–0.94
Paraffin	0.81	0.67–0.97
Gas	2.04	1.03–6.48
No. of siblings	1.06	1.01–1.13
No. of carers in household	0.94	0.89–0.99
No. of children aged <10 years in household	1.08	1.01–1.16
No. of smokers in household	1.11	0.97–1.30
Mother positive of carriage around time of birth	1.58	1.06–2.06
Type of sample: Cord vs. venous blood (ref.)	0.82	0.69–0.98
Month of swab		
January	Ref.	
February	1.22	0.76–1.87
March	1.22	0.78–1.94
April	1.12	0.72–1.64
May	1.02	0.63–1.53
June	1.95	1.08–2.92
July	2.57	1.37–3.64
August	2.19	1.13–3.17
September	1.99	1.04–2.71
October	1.93	1.06–2.79
November	1.84	1.01–3.27
December	0.88	0.55–1.32
CbpA	1.17	1.00–1.35
LytC	1.17	1.01–1.32
PcpA	1.13	1.00–1.24
PhtD1	0.79	0.70–0.95
PiaA	1.16	1.02–1.27
PspAFam2	0.85	0.77–0.98
PspAFam1	1.07	0.99–1.19
RrgBT4	1.15	1.05–1.25
Spr0096	0.92	0.85–1.00
StkP	0.88	0.81–0.99

Abbreviations: CI, confidence interval; IgG, immunoglobulin G.

^**a**^The hazard ratios for the anti-protein antibodies should be interpreted as the hazard ratio per unit increase in log IgG concentration.

**Figure 3.**
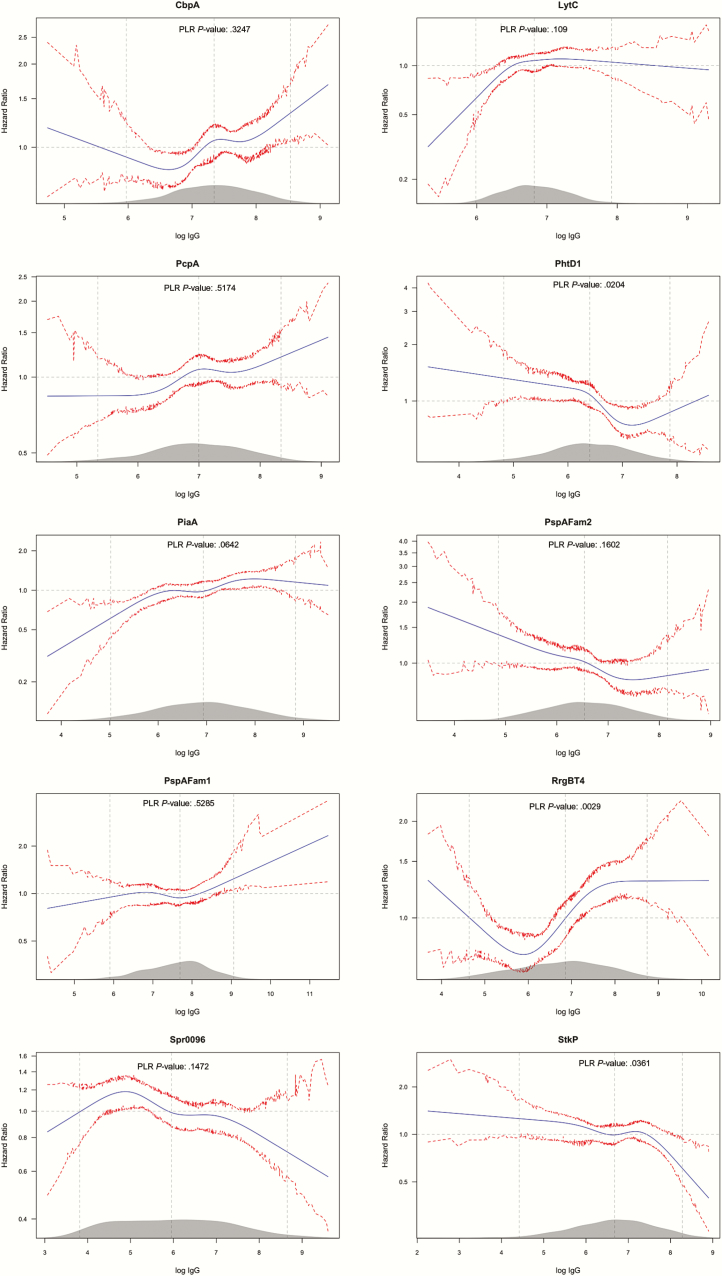
Multivariable analysis of the effect of the selected anti-protein IgG on all pneumococcal carriage acquisition rates. The figure follows the convention described in the legend for Figure 1. The 95% CIs are percentile-based and computed from 200 cluster bootstrap (clustered on subject/individual) resamples of the data. PLR stands for penalized likelihood ratio test, comparing a model with the log IgG concentration variable and one without. Abbreviations: CI, confidence interval; IgG, immunoglobulin G.

Among the 10 proteins in which adjusted nonlinear effects on carriage acquisition were analyzed, higher concentrations of PhtD1 and StkP were associated (*P* = .020 and 0.036, respectively) with reduced acquisition. Higher concentrations of RrgBT4 were associated (*P* = .003) with an increase in acquisition rate for the greater part of the distribution of anti-RrgBT4 antibodies (Figure 3). The function for anti-RrgBT4 concentration was nonmonotonic; we also observed higher carriage rates in the lower tail of the distribution. The effects of the anti-protein concentration on acquisition of any of the 5 serotypes without adjusting for the anticapsular concentrations (Figure S2) were similar with adjustment (Figure S3).

**Figure 4.**
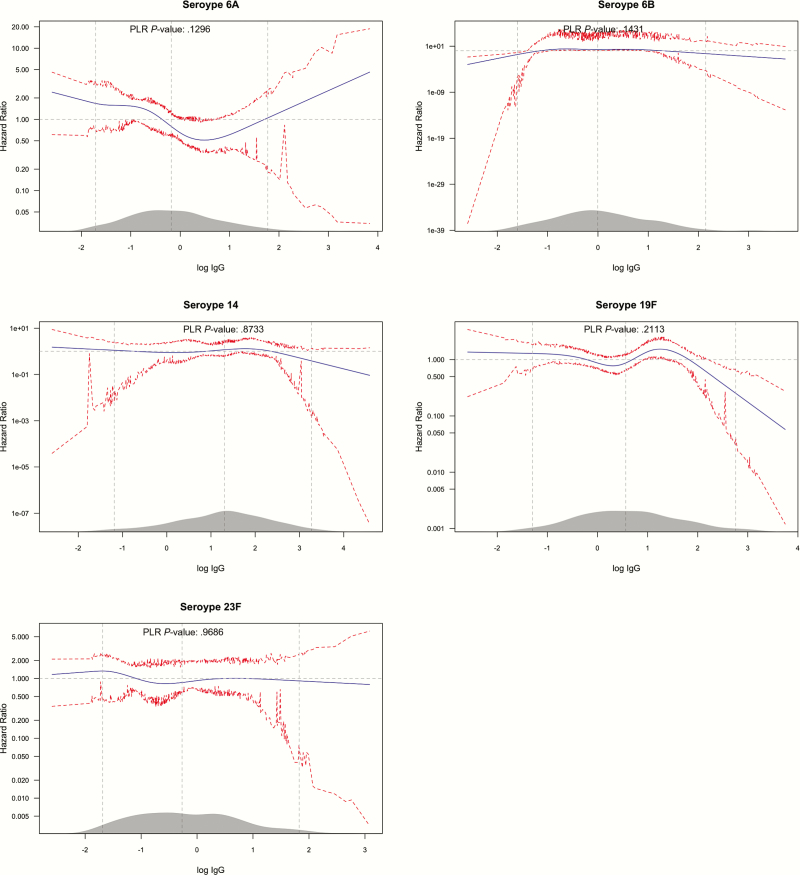
Multivariable analysis of the effect of anticapsular IgG on serotype-specific carriage acquisition rate. The figure follows the convention described in the legend for Figure 1. The 95% CIs are percentile-based and computed from 200 cluster bootstrap (clustered on subject/individual) resamples of the data. PLR stands for penalized likelihood ratio test, comparing a model with the log IgG concentration variable and one without. Abbreviations: CI, confidence interval; IgG, immunoglobulin G.

Adjusting for environmental and anti-protein antibody variables in the models of the effect of serotype-specific anticapsular antibodies only marginally influenced the shape of the hazard functions compared to the unadjusted analyses. In serotype 19F there was lower risk in the upper tail of the distribution of IgG concentration; however, there was no evidence an overall effect of serotype-specific IgG (*P* = .13–0.97) (Figure 4). In the joint multivariable analysis of all 5 serotypes, individuals with standardized log IgG concentration of about 2 and above had significant reduction in the rate of acquisition, by 50% or more, compared to individuals with the average standardized log IgG (Figure 2, right panel). A standardized log IgG concentration of 2 is equivalent to an absolute IgG concentration of 11.1, 9.8, 3.1, 5.5, and 19.0 mcg/mL for serotypes 6A, 6B, 14, 19F, and 23F, respectively.

## DISCUSSION

We assessed the association between maternally derived anti-protein and anticapsular antibodies and the rate of carriage-acquisition in the first 90 days of life among mother-infant pairs in the prevaccination period in Kenya. Among anti-protein antibodies, we found that higher concentrations of antibody to some proteins (PhtD1, PspAFam2, Spr0096, and StkP) were associated with a reduction in the acquisition rate but, unexpectedly, other proteins (CbpA, LytC, PcpA, PiaA, PspAFam1, and RrgBT4) were associated with an increase in acquisition rate (Figure 3).

Some of the associations are consistent with our understanding of the protein functions. For instance, higher anti-protein antibodies to histidine triad protein (PhtD) were significantly associated with a reduction in acquisition. PhtD is a pneumococcal cell surface protein that contributes to the adherence of *S. pneumoniae* to epithelial cells [34]. This protein has been used as a vaccine candidate [35].

Several associations are apparently inconsistent with known functions of the proteins. The presence of pilus has been implicated in adhesion to epithelial cells in humans and mice [36], suggesting a role in colonization. RrgB is a backbone subunit of pneumococcus pilus-1; anti-RrgBT4 antibody binding to pilus might reduce its capacity to bind epithelial cells and thereby abrogate its role in adhesion. Therefore, higher anti-RrgBT4 concentrations would result in lower carriage acquisition. The pneumococcal serine threonine protein kinase (StkP) has recently been shown to repress the expression of pilus and modulate bacterial adherence to human epithelial cells [37]. Thus, higher anti-StkP antibodies imply impaired repression of the pilus in the pneumococcus enhancing the attachment capacity of the bacterium, leading to increased carriage acquisition.

However, we observed a nonmonotonic relation between carriage acquisition anti-RrgBT4 concentrations; the hazard was higher at the lower and upper extremes of the concentration scale. We also observed a significant reduction in acquisition rate with increasing anti-StkP concentrations (Figure 3). The pilus is only expressed in 30–50% of pneumococci [38]; therefore, any effect mediated through pilus is likely to be diluted, thus the observed effect of anti-RrgBT4 and anti-StkP antibodies might not be entirely explained through their effect on the pilus. We did not determine pilus phenotypes of pneumococci isolated in this study, so we were unable to include the phenotype in our analysis. Nonetheless, StkP is a global kinase involved in regulating a number of pneumococcal functions that are critical for the resistance of pneumococcus to various stress conditions; one such function is cell wall biosynthesis [39]. Antibodies to StkP may inhibit the role it plays in cell wall development, and that would lead to a decrease in pneumococcal viability and therefore reduced carriage. Some of these paradoxical associations may be due to the observational nature of the study, where it is difficult to control completely for confounding; the findings could be tested in an experimental design using nasopharyngeal challenge studies in animal models. Understanding both the positive and negative impacts of antibodies on adhesion may be useful for vaccine design.

Several prior studies have documented the limited role of maternally derived antibodies in protecting infants from pneumococcal colonization [21, 22, 40]. Maternal vaccination in the third trimester of pregnancy increases the amount of antibody passed on to the newborn [20, 41]. However, there is insufficient evidence that maternal vaccination during pregnancy could reduce infant carriage or infections [42].

We observed a reduction in the rate of acquisition associated with high levels of maternally derived anticapsular antibodies to 5 serotypes even though none of the associations was statistically significant across the whole range of concentrations (Figure 2 and 4). The limited effect of anticapsular antibodies on carriage acquisition rates in our study could suggest that these antibodies are not effective against carriage acquisition, at least in an environment such as Kilifi with a high force of infection. However, we did observe a steep reduction in acquisition of carriage at very high concentration of antibodies in the range of 3.1–19.0 mcg/mL across different serotypes, though concentrations this high were attained by only about 3% of the newborn population (Figure 2). In other settings the proportion attaining these concentrations at birth might be higher; about 25% of newborns had these level of antibodies for serotypes 14 and 19F in a study of American children [43]. This provides some rationale for maternal vaccination, if the vaccine can raise the level of transferred antibodies to very high concentrations above those observed as a consequence of repeated natural exposure.

There are few analyses of CoP against carriage acquisition for anticapsular antibodies. A value of 5 mcg/mL was associated with protection against carriage of serotype 14 [17]. Our analysis suggests that the CoP is likely to be very high, in the range 3.1–19.0 mcg/mL. In the only reported study of maternal vaccination with a PCV, the geometric mean concentrations (GMC) of anticapsular antibodies in cord-blood of newborns to vaccinated mothers were between 3 and 19 times higher, depending on serotype, compared to newborns to mothers who received a placebo. The GMC in cord-blood among newborns from vaccinated mothers ranged between 2.4 and 14.3 mcg/mL [13]. This increases the potential of PCVs for use in maternal vaccination.

The serotype-specific rates of acquisition observed among the subset of newborns whose cord or maternal venous blood were collected were very similar to the total sample of 1400 newborns that constituted the original study [1] suggesting that the subset analyzed was representative (Table 1). The newborns born in hospital had lower rates of pneumococcal acquisition compared to those born at home. The blood type (cord or venous) provided was therefore a potential confounder in the relationship between antibodies and carriage acquisition. However, we adjusted for blood type in all multivariable models.

In conclusion, we observed a significant association between carriage acquisition and several anti-protein antibodies but only a limited role for maternally derived anticapsular antibodies, at high concentrations, on serotype-specific acquisition of pneumococci. This disparity between anti-protein and anticapsular effects may be attributable to differential study power, as the anticapsular analyses was restricted to homologous acquisitions but the anti-protein analysis included all acquisitions. Nonetheless, a strategy of maternal vaccination to improve the level of transferred antibodies and thus protect newborns against acquisition of carriage may be successful if vaccine formulation is focused on enhancing specific anti-protein antibodies that are associated with reduced carriage or if the strategy induces very high concentrations of anticapsular antibodies, above those normally observed in physiological trans-placental transfer.

## Supplementary Data

Supplementary materials are available at *Clinical Infectious Diseases* online. Consisting of data provided by the authors to benefit the reader, the posted materials are not copyedited and are the sole responsibility of the authors, so questions or comments should be addressed to the corresponding author.

## Supplementary Material

Supplementary Figure S1

Supplementary Figure S2

Supplementary Figure S3

Supplementary Figure Legends

## Notes

***Acknowledgments.*** The study was approved by the Kenya Medical Research Institute (KEMRI) Ethical review committee (Protocol number, SCC 2273). This paper is published with the permission of the Director, Kenya Medical Research Institute. We thank Nicola Green for performing the pneumococcal serology.

***Financial support.*** The study was funded by the Wellcome Trust, through research fellowships [098532 to J. A. G. S. and 092767 to J. O.].

***Potential conflicts of interest.*** D. G. is funded by the NIHR, and his laboratory undertakes collaborative and contract research for GSK who make PCV. D. G. attends occasional advisory boards convened by GSK. All authors have submitted the ICMJE Form for Disclosure of Potential Conflicts of Interest. Conflicts that the editors consider relevant to the content of the manuscript have been disclosed.
