# Supplementary figures and images for: Effect of Maternally Derived Anti-protein and Anticapsular IgG Antibodies on the Rate of Acquisition of Nasopharyngeal Carriage of Pneumococcus in Newborns
Source: Clin Infect Dis. 2017 Aug 17;66(1):121–30. doi: 10.1093/cid/cix742 (PMC5850545; doi:10.1093/cid/cix742)

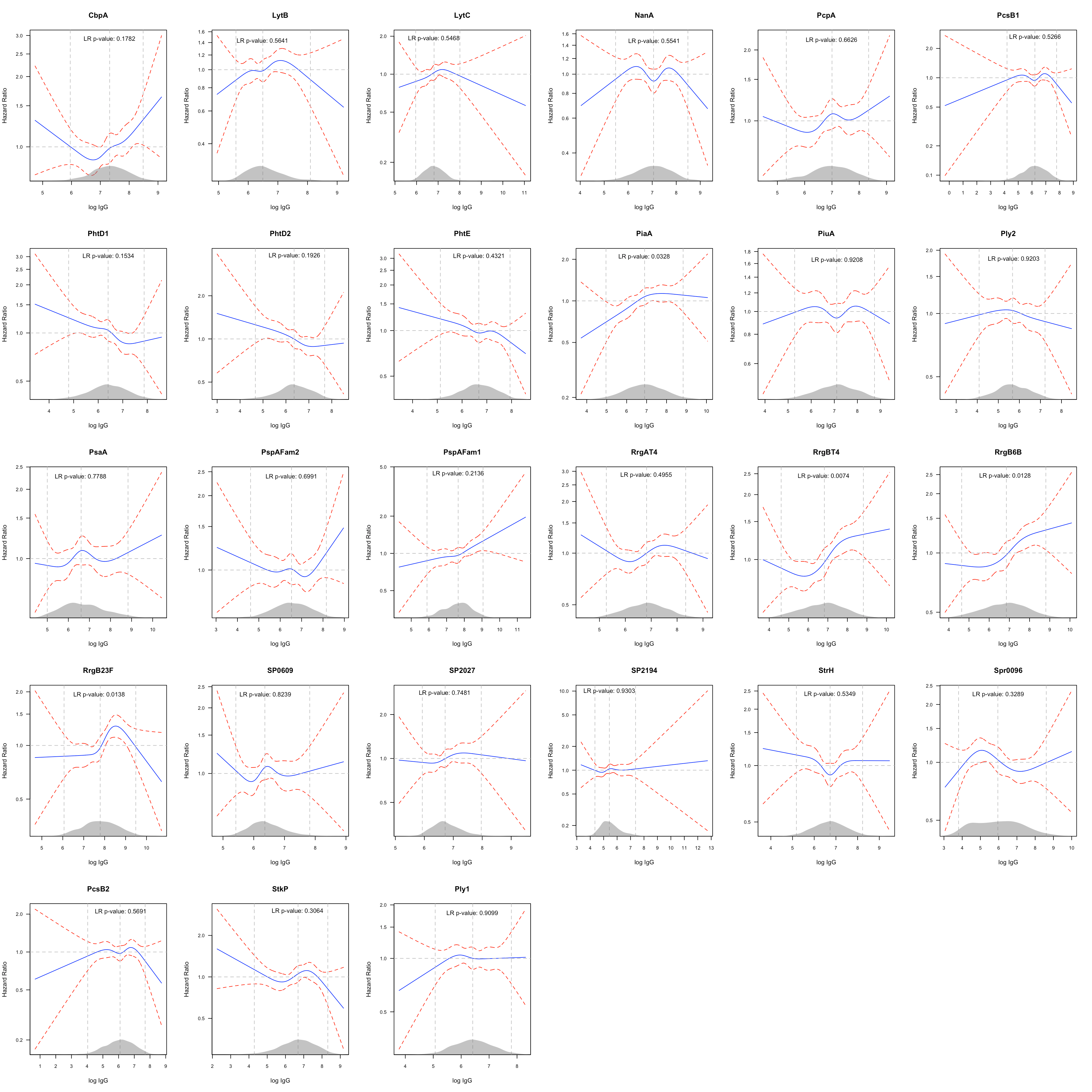

Supplement: Supplementary Figure S1 [file cix742_suppl_supplementary_figure_s1.png]

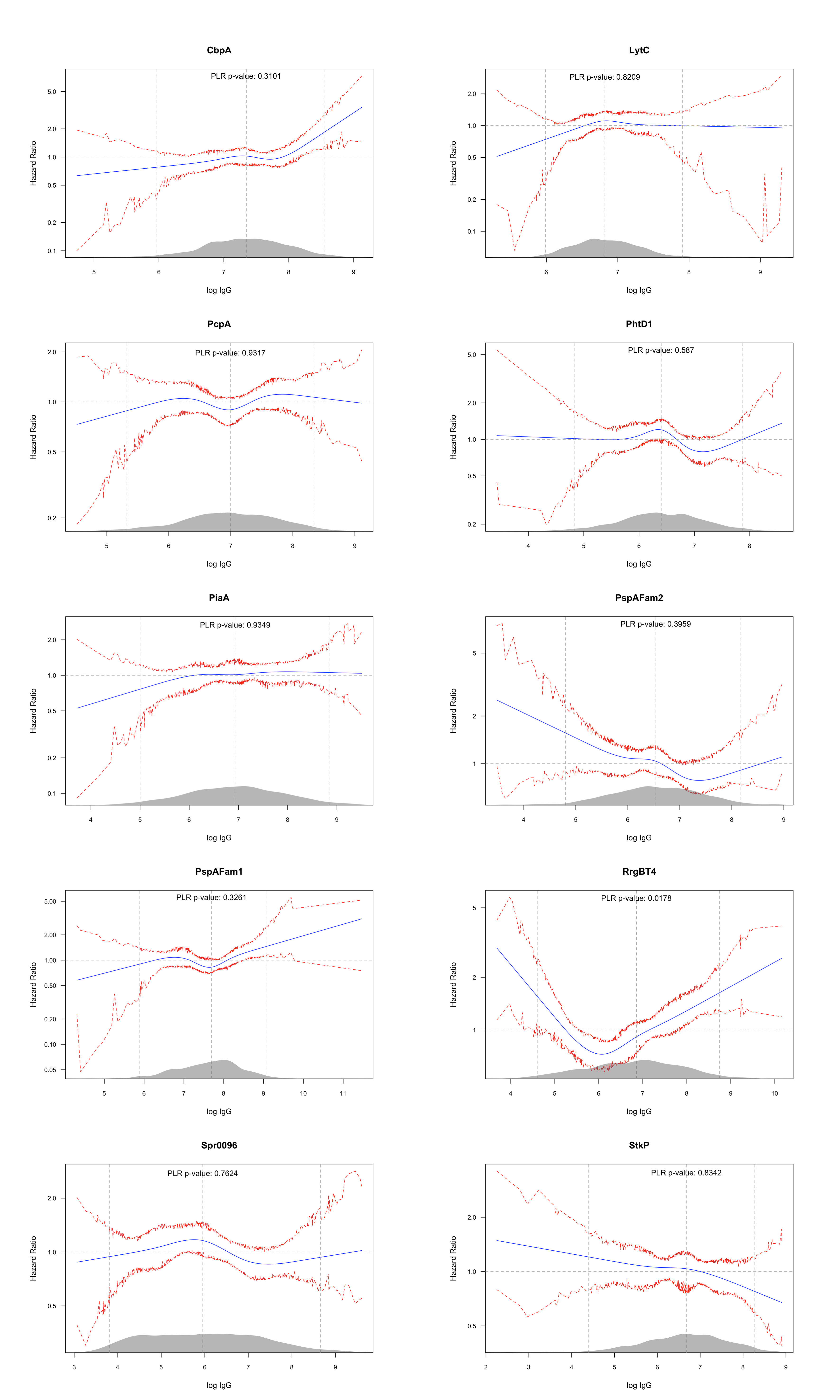

Supplement: Supplementary Figure S2 [file cix742_suppl_supplementary_figure_s2.png]

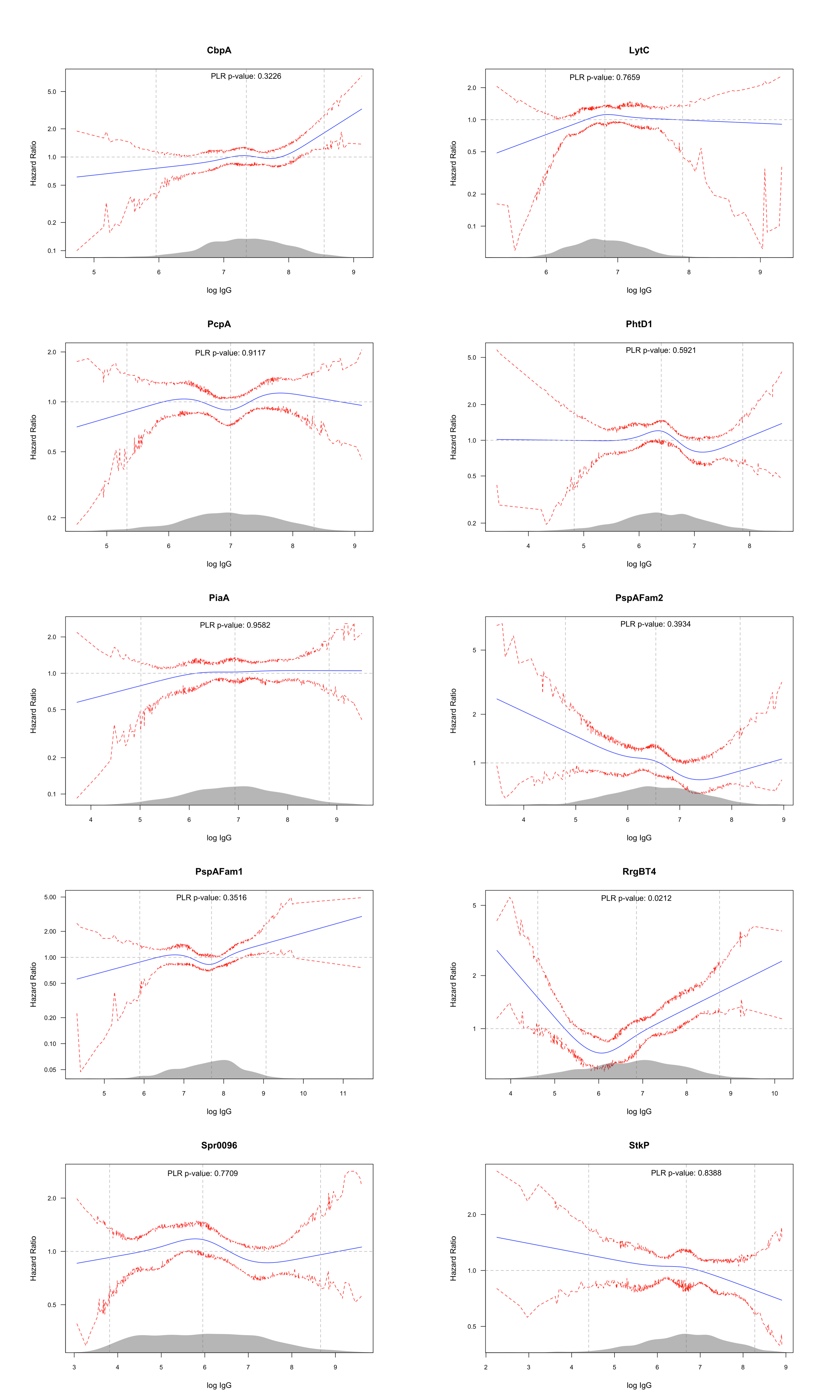

Supplement: Supplementary Figure S3 [file cix742_suppl_supplementary_figure_s3.png]
