# Supplementary material for: Effect of Maternally Derived Anti-protein and Anticapsular IgG Antibodies on the Rate of Acquisition of Nasopharyngeal Carriage of Pneumococcus in Newborns
Source: Clin Infect Dis. 2017 Aug 17;66(1):121–30. doi: 10.1093/cid/cix742 (PMC5850545; doi:10.1093/cid/cix742)
Supplement: Supplementary Figure Legends [file cix742_suppl_supplementary_figure_legends.pdf]

Supplementary Figure Legends

Supplementary Figure S1: Univariable analysis of the effect of anti-protein IgG concentration on all pneumococcal carriage acquisition rates. The figure follows the convention described in the legend for figure 1.

Supplementary Figure S2: Multivariable analysis of the effect of anti-protein IgG concentration on carriage acquisition rates of any of the serotypes: 6A, 6B, 14, 19F and 23F not adjusting for anti-capsular antibodies. Adjustment variables for the functions presented in the plot include the environmental variables and rest of the selected anti-protein variables. The figure follows the convention described in the legend for figure 1.

Supplementary Figure S3: Multivariable analysis of the effect of anti-protein IgG concentration on carriage acquisition rates of any of the serotypes: 6A, 6B, 14, 19F and 23F, adjusting for standardized anti-capsular antibodies. Adjustment variables included the environmental variables, the rest of the selected anti-protein variables and standardized log anti-capsular antibodies of the five serotypes. The figure follows the convention described in the legend for figure 1.
